# Development of a nomogram to predict surgical site infection after closed comminuted calcaneal fracture

**DOI:** 10.1186/s12893-022-01735-4

**Published:** 2022-08-12

**Authors:** Jia-sen Hu, Cheng-bin Huang, Shu-ming Mao, Kang-hao Fang, Zong-yi Wu, You-ming Zhao

**Affiliations:** 1 Department of Orthopaedic Surgery, The Second Affiliated Hospital and Yuying Childrens Hospital of Wenzhou Medical University, Wenzhou, 325000 China; 2 Key Laboratory of Orthopaedics of Zhejiang Province, Wenzhou, 325000 China

**Keywords:** Closed comminuted calcaneal fractures, Surgical site infection, Risk factor, Nomogram model

## Abstract

**Background:**

Compared with open comminuted calcaneal fractures, less emphasis is placed on postoperative surgical site infection (SSI) of closed comminuted calcaneal fractures. This study aimed to identify the risk factors associated with SSI and build a nomogram model to visualize the risk factors for postoperative SSI.

**Methods:**

We retrospectively collected patients with closed comminuted calcaneal fractures from the Second Affiliated Hospital of Wenzhou Medical University database from 2017 to 2020. Risk factors were identified by logistics regression analysis, and the predictive value of risk factors was evaluated by ROC (receiver operating characteristic curve). Besides, the final risk factors were incorporated into R4.1.2 software to establish a visual nomogram prediction model.

**Results:**

The high-fall injury, operative time, prealbumin, aspartate aminotransferase (AST), and cystatin-C were independent predictors of SSI in calcaneal fracture patients, with OR values of 5.565 (95%CI 2.220–13.951), 1.044 (95%CI 1.023–1.064), 0.988 (95%CI 0.980–0.995), 1.035 (95%CI 1.004–1.067) and 0.010 (95%CI 0.001–0.185) (P_s_ < 0.05). Furthermore, ROC curve analysis showed that the AUC values of high-fall injury, operation time, prealbumin, AST, cystatin-C, and their composite indicator for predicting SSI were 0.680 (95%CI 0.593–0.766), 0.756 (95%CI 0.672–939), 0.331 (95%CI 0.243–0.419), 0.605 (95%CI 0.512–0.698), 0.319 (95%CI 0.226–0.413) and 0.860 (95%CI 0.794–0.926), respectively (P_s_ < 0.05). Moreover, the accuracy of the nomogram to predict SSI risk was 0.860.

**Conclusions:**

Our study findings suggest that clinicians should pay more attention to the preoperative prealbumin, AST, cystatin C, high-fall injury, and operative time for patients with closed comminuting calcaneal fractures to avoid the occurrence of postoperative SSI. Furthermore, our established nomogram to assess the risk of SSI in calcaneal fracture patients yielded good accuracy and can assist clinicians in taking appropriate measures to prevent SSI.

## Introduction

Fracture is a relatively common disease in the world. Different surgical methods are often adopted for fracture of different parts, such as external fixation bracket for pelvic fracture and internal fixation plate for humeral shaft fracture [1]. However, patients are often susceptible to postoperative complications irrespective of the treatment approach. One of the most severe complications is surgical site infection (SSI). SSIs are widely acknowledged to impair surgical incision healing and even lead to life-threatening osteomyelitis [2].

Comminuted calcaneal fractures are more prone to SSI due to their specific location and severe soft tissue injury [3]. Clinicians often pay more attention to postoperative surgical site infection for open fractures, given that they have a higher risk of surgical site infection than closed ones [4]. However, little emphasis has been placed on the perioperative situation of patients with closed fractures.

Therefore, this study sought to analyze factors associated with postoperative surgical site infection in patients with a closed comminuted calcaneal fracture by analyzing the preoperative clinical characteristics, laboratory parameters, and surgical records. Although several studies [5–7] have examined the risk factors for postoperative infection in patients with comminuted calcaneal fractures, this is the first study to build a nomogram model to visualize the risk factors for postoperative surgical site infection in patients with closed comminuted calcaneal fractures. It is widely acknowledged that a nomogram is a computational diagram that can replace complex mathematical formulas and integrate more clinical variables to make accurate individual predictions [8]. In addition, it provides a repeatable and straightforward tool to predict the risk of SSI in patients with calcaneal fractures, unlike other studies that provide a mathematically more complicated model. In a nutshell, we designed this study to investigate the risk factors for postoperative SSI in patients with calcaneal fracture and establish a nomogram prediction model.

## Methods

### Study design

With the approval of the Institutional Review Committee, we retrospectively collected patients with unilateral closed comminuted calcaneal fractures from the database of the Second Affiliated Hospital of Wenzhou Medical University from 2017 to 2020. A total of 214 patients with unilateral closed comminuted calcaneal fractures were included in this study and divided into two groups based on the incidence of surgical site infection: the SSI (Surgical site infection) group and the non-SSI group. Preoperative clinical characteristics, laboratory parameters, and operative records were collected for all patients. These baseline variables included age, body mass index (BMI), injury mechanism, injury-surgery interval, operative time, operative blood loss, gender, types of admission, current drinking, current smoking, injured feet, artificial bone graft, surgical approach, anesthesia method, preoperative mannitol, postoperative antibiotic, postoperative drainage tube, hypertension, diabetes, fatty liver, kidney stone, pneumonia, venous thrombus embolism (VTE), education level, prealbumin, total protein, albumin, globulin, albumin/globulin (A/G), aspartate aminotransferase (AST), alanine aminotransferase (ALT), ASL/ALT, alkaline phosphatase (ALP), gamma-glutamyltransferase (GGT), total bilirubin, direct bilirubin, indirect bilirubin, fasting blood glucose (FBG), blood urea nitrogen (BUN), serum creatinine (Scr), BUN/Scr, cystatin-C, creatine phosphate kinase (CPK), homocysteine, lactate dehydrogenase (LDH), cholinesterase, blood uric acid, serum sodium, serum kalium, serum calcium, serum chlorine, white blood cell (WBC), neutrophils, lymphocyte, monocyte, eosinophilic granulocyte, basophilic granulocyte, red blood cell (RBC), hemoglobin, hematocrit, mean corpuscular volume (MCV), mean corpuscular hemoglobin (MCH), mean corpusular hemoglobin concerntration (MCHC), platelet count, plateletcrit, platelet distribution width (PDW) and mean platelet volume (MPV).

### The definition of SSI

SSI diagnosis after surgery was based on CDC's National Healthcare Surveillance Network (NHSN) [9, 10]. Superficial SSI mainly involves the skin or subcutaneous tissue of the incision, and its diagnosis requires at least one of the following: (1) purulent exudate from the superficial incision, (2) positive microbial culture of the superficial exudate, and (3) redness, swelling, heat and pain of the incision. Deep SSI mainly involves deep soft tissue, and its diagnosis requires at least one of the following: (1) purulent exudate from the deep incision, (2) local abscess requiring debridement or plate removal, (3) positive microbial culture of deep exudate, (4) abscesses or other signs of infection detected by radiological or histopathological examination.

### Inclusion and exclusion criteria

The inclusion criteria were: (1) Unilateral comminuted calcaneal fracture, (2) No other fractures, 3) age > 18 years, (4) The diagnostic criteria for SSI were in accordance with clinical guidelines. The exclusion criteria were: (1) open fracture, (2) with other fractures, (3) pathological fracture, (4) missing clinical characteristics or laboratory parameters.

### Statistics

The normality of the data distribution was tested using the Shapiro-Wilk test. Patient characteristics were described using median (interquartile range [IQR]) and mean ± standard deviation, frequency, and percentage when appropriate. A nonparametric test (Mann-Whitney U test or Kruskal-Wallis test) was applied for data with non-normal distribution or heterogeneity of variances. Categorical variables were expressed as percentages and analyzed using the Pearson Chi-squared test. Univariate logistic regression analysis was used to determine the independent risk factors for incision infection. Moreover, risk factors significantly associated with SSI in the univariate analysis (P < 0.1) were included in the multivariable logistic regression. Receiver operating characteristic (ROC) curves were applied to analyze predictive value indicators for closed comminuted calcaneal fracture patients with SSI. All statistics were calculated using SPSS software (version 26.0; SPSS Inc., Chicago, IL, USA). Besides, the final risk factors were incorporated into R4.1.2 software (R Foundation for Statistical Computing, Vienna, Austria) to establish a nomogram prediction model. The consistency index (C-index) was used to evaluate the model's prediction performance, and the correction curve was used to judge the prediction consistency [11]. The range of the C-index value was 0.5 to 1.0, and accuracy was positively correlated with the value. The calibration curve included an image. The calibration curve included an image comparison of predicting risk and SSI risk. The closer the predicted risk to the standard curve, the better the conformity of the model.

## Results

### Baseline characteristics of the study population

214 patients were enrolled in this study, including 44 in the SSI group and 170 in the non-SSI group. There were statistically significant differences between the two groups in operative blood loss, operative time, injury mechanism, injury-surgery interval, types of admission, surgical approach, prealbumin, A/G, AST, cystatin-C, CPK, cholinesterase, and eosinophilic granulocyte (P_s_ < 0.05). Moreover, there were no statistically significant differences between the two groups in BMI, education level, gender, current drinking, current smoking, injured feet, artificial bone graft, anesthesia method, preoperative mannitol, postoperative antibiotic, postoperative drainage tube, hypertension, diabetes, fatty liver, pneumonia, kidney stones, VTE, total protein, albumin, globulin, ALT, ASL/ALT, ALP, GGT, total bilirubin, direct bilirubin, indirect bilirubin, FBG, BUN, Scr, BUN/Scr, homocysteine, LDH, blood uric acid, serum sodium, serum kalium, serum calcium, serum chlorine, WBC, neutrophils, lymphocyte, monocyte basophilic granulocyte, RBC, hemoglobin, hematocrit, MCV, MCH, MCHC, platelet count, plateletcrit, PDW and MPV. (Details are shown in Table 1).

**Table 1:** Comparison of preoperative clinical characteristics and preoperative laboratory parameters between two groups

	Non-SSI (170)	SSI (44)	P value
Age (years)	46.12 ± 11.45	47.25 ± 11.08	0.559
BMI	23.94 (22.24–25.49)	24.12 (22.31–25.81)	0.374
*Injury mechanism*			< 0.001
Low fall injury (< 2 m)	104 (61.2)	11 (25.0)	
High fall injury (> 2 m)	66 (38.8)	33 (75.0)	
Injury-surgery interval (days)	4 (3–5)	5 (3.25–7)	0.044
Operative time (minutes)	70 (60–81)	93 (80–104)	< 0.001
Operative blood loss (mL)	50 (20–50)	85 (50–100)	< 0.001
*Gender*			0.819
Female, n (%)	21 (12.4)	6 (13.6)	
Male, n (%)	149 (87.6)	38 (86.4)	
*Types of admissi*on			0.022
Outpatient, n (%)	79 (46.5)	12 (27.3)	
Emergency, n (%)	91 (53.5)	32 (72.7)	
Current drinking, n (%)	75 (44.1)	19 (43.2)	0.911
Current smoking, n (%)	66 (38.8)	21 (47.7)	0.284
*Injured feet*			0.317
Left, n (%)	90 (52.9)	27 (61.4)	
Right, n (%)	80 (47.1)	17 (38.6)	
Artificial bone graft, n (%)	129 (75.9)	35 (79.5)	0.609
*Surgical approach*			0.019
Tarsal sinus approach, n (%)	58 (34.1)	7 (15.9)	
Extended lateral approach, n (%)	112 (65.9)	37 (84.1)	
*Anesthesia method*			0.054
Combined spinal and epidural anesthesia, n (%)	146 (85.9)	32 (72.7)	
General anesthesia, n (%)	16 (9.4)	10 (22.7)	
Spinal anesthesia, n (%)	8 (4.7)	2 (4.5)	
Preoperative mannitol, n (%)	143 (84.1)	36 (81.8)	0.713
*Postoperative antibiotic*			0.066
First-generation cephalosporins, n (%)	6 (3.5)	4 (9.1)	
Second-generation cephalosporins, n (%)	68 (40.0)	15 (34.1)	
Third-generation cephalosporin, n (%)	13 (7.6)	5 (11.4)	
Latamoxef Sodium, n (%)	16 (9.4)	5 (11.4)	
Clindamycin, n (%)	21 (12.4)	9 (20.5)	
Azlocillin, n (%)	13 (7.6)	2 (20.5)	
Amoxicillin, n (%)	18 (10.6)	0	
Flucloxacillin, n (%)	15 (8.8)	4 (9.1)	
Postoperative drainage tube, n (%)	54 (31.8)	10 (22.7)	0.243
Hypertension, n (%)	34 (20.0))	8 (18.2	0.787
Diabetes, n (%)	14 (8.2)	3 (6.8)	0.757
Fatty liver, n (%)	72 (42.4)	13 (15.3)	0.122
Kidney stone, n (%)	27 (15.9)	3 (6.8)	0.123
Pneumonia, n (%)	20 (11.8)	5 (11.4)	0.941
*VTE*			0.218
Low-risk, n (%)	80 (47.1)	26 (59.1)	
Medium risk, n (%)	39 (22.9)	7 (15.9)	
High risk, n (%)	51 (30.0)	11 (25.0)	
*Education level*			0.521
Illiteracy, n (%)	1 (2.3)	13 (7.6)	
Primary, n (%)	14 (31.8)	49 (28.8)	
Junior middle, n (%)	19 (43.2)	78 (45.9)	
High school, n (%)	9 (20.5)	19 (11.2)	
Bachelor degree or above, n (%)	1 (2.3)	11 (6.5)	
Prealbumin (mg/L)	262.62 ± 57.32	228.70 ± 57.49	0.001
Total protein (g/L)	68.21 ± 5.39	67.86 ± 5.83	0.708
Albumin (g/L)	41.9 (39.7–44.5)	41.7 (37.6–44.1)	0.164
Globulin (g/L)	26.15 ± 3.12	26.99 ± 3.51	0.122
A/G	1.6 (1.5–1.7)	1.5 (1.4–1.7)	0.018
AST (U/L)	21.0 (18.0–26.0)	23.5 (20.0–30.8)	0.038
ALT (U/L)	21 (15–32)	23 (16–33)	0.600
AST/ALT	0.96 (0.73–1.33)	1.10 (0.82–1.44)	0.143
ALP (U/L)	72.0 (63.5–87.0)	69.0 (58.5–83.8)	0.198
GGT (U/L)	29.0 (20.0–59.5)	31.5 (18.3–50.3)	0.776
Total bilirubin (umol/L)	15.4 (11.8–20.6)	16.5 (13.5–20.1)	0.289
Direct Bilirubin (umol/L)	4.0 (2.9–5.1)	4.3 (3.6–6.0)	0.112
Indirect Bilirubin (umol/L)	11.3 (8.6–15.7)	12.9 (10.2–15.2)	0.255
FBG (mmol/L)	5.38 (4.82–6.37)	5.33 (4.88–6.43)	0.990
BUN (mmol/L)	5.30 (4.40–6.70)	4.89 (4.33–6.40)	0.270
Scr (umol/L)	65.55 ± 12.01	63.60 ± 12.19	0.338
BUN/Scr	0.08 (0.07–0.1)	0.08 (0.07–0.10)	0.736
Cystatin-C (mg/L)	0.89 (0.78–0.99)	0.79 (0.68–0.93)	< 0.001
CPK (U/L)	259 (165–442)	475 (200.25–599)	0.007
Homocysteine (umol/L)	9.60 (8.10–11.20)	10.35 (8.88–11.20)	0.180
LDH (U/L)	190 (171–212)	202 (173–233)	0.174
Cholinesterase (U/L)	8973 (7645–10,222)	8224 (7064–8650)	0.006
Blood uric acid (umol/L)	350.81 ± 98.65	318.93 ± 96.28	0.056
Serum sodium (mmol/L)	139.5 (138.2–141.0)	139.2 (137.3–141.3)	0.384
Serum kalium (mmol/L)	3.86 (3.63–4.10)	3.96 (3.78–4.17)	0.195
Serum calcium (mmol/L)	2.25 ± 0.12	2.24 ± 0.11	0.586
Serum chlorine (mmol/L)	104.89 ± 2.33	104.45 ± 2.36	0.265
WBC (10^9^/L)	8.18 (6.83–10.85)	9.36 (7.21–10.84)	0.334
Neutrophils (10^9^/L)	5.69 (4.66–8.23)	6.96 (4.83–8.60)	0.302
Lymphocyte (10^9^/L)	1.56 (1.20–1.98)	1.57 (1.17–1.97)	0.969
Monocyte (10^9^/L)	0.54 ± 0.23	0.57 ± 0.27	0.427
Eosinophilic granulocyte (10^9^/L)	0.08 (0.03–0.16)	0.04 (0.01–0.12)	0.012
Basophilic granulocyte (10^9^/L)	0.012 (0.007–0.022)	0.012 (0.008–0.198)	0.593
RBC (10^12^/L)	4.53 ± 0.53	4.47 ± 0.60	0.489
Hemoglobin (g/L)	151 (141–157)	151 (144–156.5)	0.832
Hematocrit	0.41 (0.39–0.44)	0.42 (0.38–0.44)	0.533
MCV (fl)	90.90 (88.05–93.55)	91.35 (88.55–94.28)	0.423
MCH (pg)	31 (29.8–31.9)	30.9 (30.05–32.15)	0.973
MCHC (g/L)	340.72 ± 10.56	339.43 ± 10.75	0.474
Platelet count (10^9^/L)	217.36 ± 58.50	214.18 ± 67.07	0.756
Plateletcrit	0.22 (0.19–0.26)	0.21 (0.19–0.26)	0.920
PDW, n (%)	14 (11.90–15.90)	13.35 (11.90–15.48)	0.486
MPV (fl)	10.36 ± 1.02	10.54 ± 1.15	0.304

BMI body mass index; VTE venous thrombus embolism; WBC, white blood cell; RBC, red blood cell; MPV, mean platelet volume; AST, aspartate aminotransferase; ALT, alanine aminotransferase; A/G, albumin/globulin, CPK, creatine phosphate kinase; BUN, blood urea nitrogen; Scr, serum creatinine; MCHC, mean corpusular hemoglobin concerntration; ALP, alkaline phosphatase; GGT, gamma-glutamyltransferase; FBG, fasting blood glucose; LDH, lactate dehydrogenase; MCV, mean corpuscular volume; PDW, platelet distribution width; MCH, mean corpuscular hemoglobin

### Logistic regression analysis for independent risk factors of SSI in calcaneal fracture patients

The univariate logistics regressions analysis was applied to the baseline variables, laboratory tests, and comorbidities. Age, BMI, injury-surgery interval, injury mechanism, operative time, operative blood loss, gender, types of admission, current drinking, current smoking, injured feet, artificial bone graft, surgical approach, anesthesia method, preoperative mannitol, postoperative antibiotic, postoperative drainage tube, hypertension, diabetes, fatty liver, kidney stone, pneumonia, VTE, education level, prealbumin, total protein, albumin, globulin, A/G, AST, ALT, ASL/ALT, ALP, GGT, total bilirubin, direct bilirubin, indirect bilirubin, FBG, BUN, Scr, BUN/Scr, cystatin-C, CPK, homocysteine, LDH, cholinesterase, blood uric acid, serum sodium, serum kalium, serum calcium, serum chlorine, WBC, neutrophils, lymphocyte, monocyte, eosinophilic granulocyte, basophilic granulocyte, RBC, hemoglobin, hematocrit, MCV, MCH, MCHC, platelet count, plateletcrit, PDW and MPV were analyzed during the univariate analysis. Parameters significantly associated with SSI, including injury-surgery interval, high-fall injury, operative time, operative blood loss, types of admission, surgical approach, prealbumin, albumin, A/G, AST, total bilirubin, direct bilirubin, cystatin-C, CPK, LDH, cholinesterase, blood uric acid, and eosinophilic granulocyte (P < 0.1) (Table 2) were included in multiple logistic regression analysis. The results showed that the high-fall injury, operative time, prealbumin, AST, and cystatin-C were independent predictors of SSI in calcaneal fracture patients, with OR values of 5.565 (95%CI 2.220–13.951), 1.044 (95%CI 1.023–1.064), 0.988 (95%CI 0.980–0.995), 1.035 (95%CI 1.004–1.067) and 0.010 (95%CI 0.001–0.185) (P_s_ < 0.05) (Table 3).

**Table 2:** Univariate logistics regressions analysis of risk factors to closed comminuted calcaneal fracture patients with surgical site infection

Variables	OR	95%CI	P
Injury-surgery interval (days)	1.116	0.992–1.256	0.068
High-fall injury	4.727	2.236–9.996	< 0.001
Operative time (minutes)	1.042	1.024–1.060	< 0.001
Operative blood loss (ml)	1.011	1.003–1.018	0.005
Types of admission	2.315	1.117–4.798	0.024
Surgical approach	2.737	1.149–6.519	0.023
Prealbumin (mg/L)	0.990	0.984–0.996	0.001
Albumin (g/L)	0.919	0.853–0.990	0.026
A/G	0.196	0.042–0.914	0.038
AST (U/L)	1.026	1.002–1.050	0.031
Total bilirubin (umol/L)	1.031	0.995–1.068	0.097
Direct Bilirubin (umol/L)	1.162	1.024–1.319	0.020
Cystatin-C (mg/L)	0.007	0.001–0.092	< 0.001
CPK (U/L)	1.001	1.000–1.002	0.022
LDH (U/L)	1.007	1.000–1.015	0.059
Cholinesterase (U/L)	1.000	1.000–1.000	0.017
Blood uric acid (umol/L)	0.997	0.993–1.000	0.058
Eosinophilic granulocyte (10^9/L)	0.008	0.001–0.553	0.026

AST, aspartate aminotransferase; A/G, albumin/globulin, CPK, creatine phosphate kinase; LDH, lactate dehydrogenase

**Table 3:** Multivariate logistics regressions analysis of risk factors to closed comminuted calcaneal fracture patients with surgical site infection

Variables	OR	95%CI	P
High-fall injury	5.565	2.220–13.951	< 0.001
Operative time (minutes)	1.044	1.023–1.064	< 0.001
Prealbumin (mg/L)	0.988	0.980–0.995	0.001
AST (U/L)	1.035	1.004–1.067	0.027
Cystatin-C (mg/L)	0.010	0.001–0.185	0.002

AST, aspartate aminotransferase

Receiver operating characteristic curve (ROC) analysis was performed to assess the predictive value of the composite indicators of SSI in calcaneal fracture patients.

**Fig. 1:**
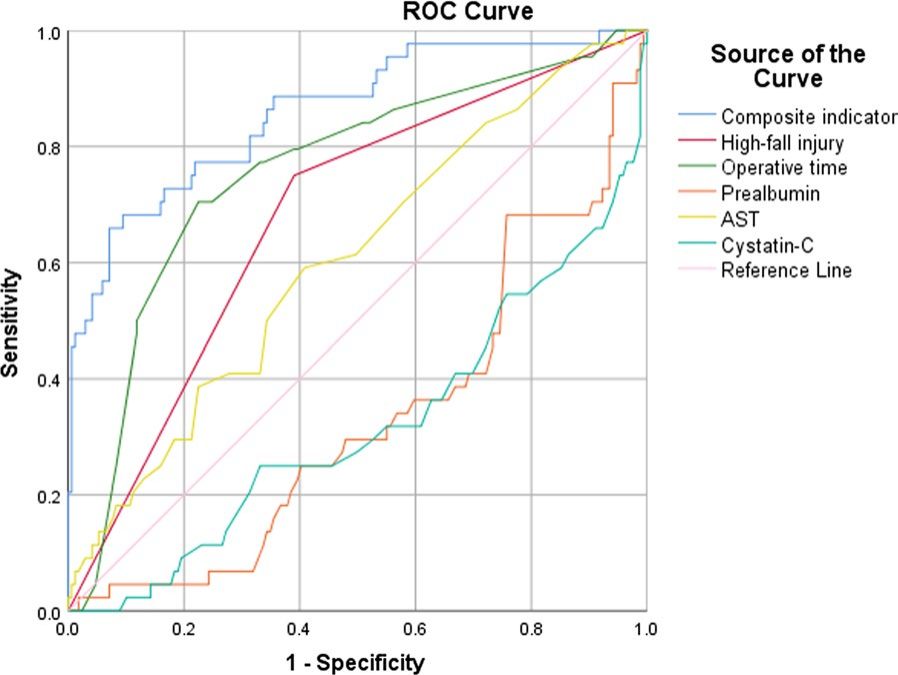
Discriminatory accuracy for predicting calcaneal fracture patients with SSI by receiver operator characteristics (ROC) analysis calculating area under the curve (AUC)

Taking the occurrence of SSI as the status variable and high-fall injury + operative time + prealbumin + AST + cystatin-C as test variables of the composite indicator, ROC curve analysis showed yielded an Area Under Curve (AUC) value of 0.860 (95%CI 0.794 -0.926, P < 0.001) for composite indicators for the prediction of SSI. In addition, the AUC of high-fall injury, operation time, prealbumin, AST and cystatin-C was 0.680 (95%CI 0.593–0.766), 0.756 (95%CI 0.672–939), 0.331 (95%CI 0.243–0.419), 0.605 (95%CI 0.512–0.698) and 0.319 (95%CI 0.226–0.413) (P_s_ < 0.05), respectively (Fig. 1).

**Fig. 2:**
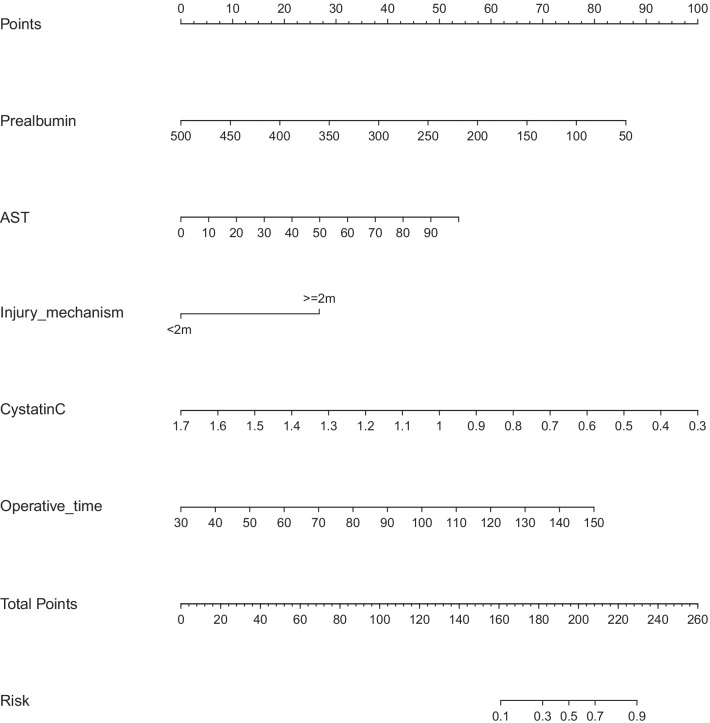
A nomogram to predict the incidence of SSI in calcaneal fracture patients

A nomogram was established to predict the risk of SSI (Fig. 2).

**Fig. 3:**
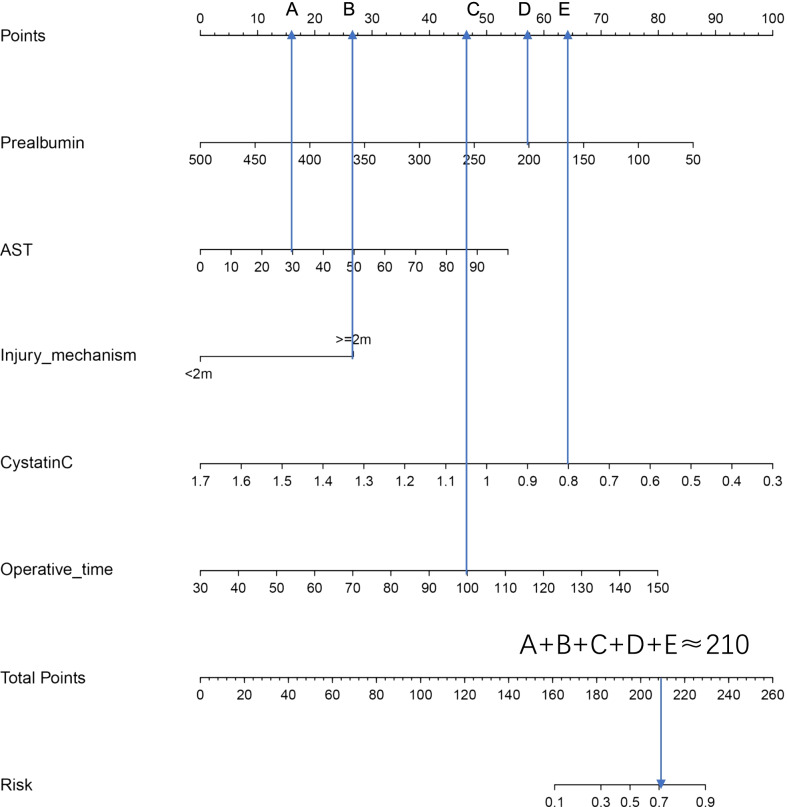
Example of using nomogram to predict SSI

**Fig. 4:**
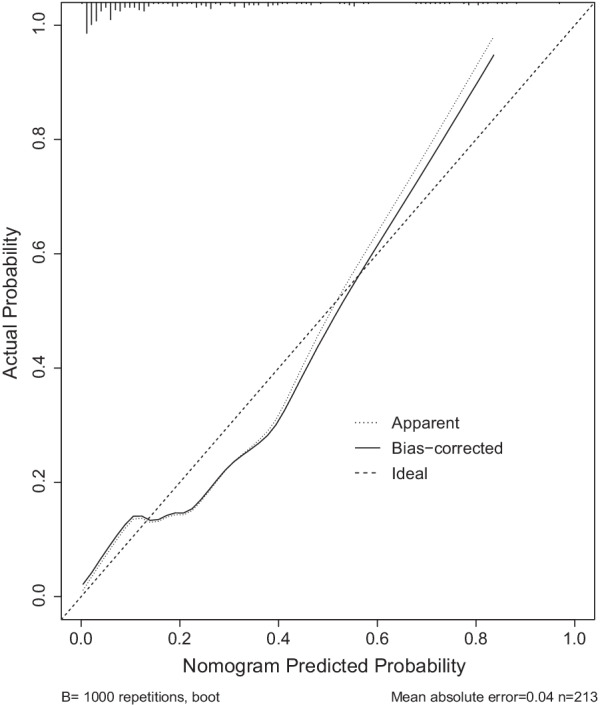
Calibration curve for nomogram prediction of SSI in calcaneal fracture patients

The total scores of every single item, while the probability of SSI in calcaneal fracture patients is obtained by the total score. For example, for a patient with a unilateral closed comminuted calcaneal fracture, preoperative prealbumin of 200 g/L, AST of 30 and cystatin-C of 0.8, fracture caused by a fall from a height of 2 m, and an operative time of 100 min, the probability of SSI was approximately 70% (Fig. 3). The C-index of the model was 0.860 after 1000 bootstrap self-sampling replicates, which indicated that the consistency between the predicted value and the actual observation value is by the standard and has a standard resolution. Furthermore, the coefficient of determination (R^2^) of the calibration curve (Fig. 4) was 0.443, suggesting that the curve was an excellent fit.

## Discussion

This study included 214 patients with unilateral closed comminuted calcaneal fractures from 2017 to 2020, 40 of whom developed postoperative surgical site infection. In addition, we comprehensively analyzed the preoperative clinical characteristics, laboratory parameters, and surgical records of patients with a calcaneal fracture to explore the risk factors for postoperative SSI to prevent the occurrence of SSI in this patient population. We found that preoperative prealbumin, AST, cystatin-C, high fall injury, and longer operative time were strongly associated with postoperative SSI for closed comminuted calcaneal fractures. Moreover, this composite indicator (high-fall injury + Operative time + prealbumin + AST + cystatin-C) exhibited good performance for predicting postoperative SSI in patients before surgery. To the best of our knowledge, this study is the first documented nomogram model to predict the incidence of SSI in calcaneal fracture patients to assist clinicians in better preventing the occurrence of SSI in calcaneal fracture.

High-level falls are high-energy injuries, causing severe damage to the calcaneus and surrounding soft tissue. Combined with secondary trauma to the patient during surgery, these injuries severely affect the blood supply around the calcaneus and provide a favorable microenvironment for bacterial growth at the surgical site, resulting in SSI [12], suggesting that high energy injury is a high-risk factor for SSI after calcaneal fracture. In addition, calcaneal fractures caused by high-energy injuries tend to be more complex and take longer to operate. Interestingly, Cheng H et al. [13], showed that prolonged operative time increases the risk of SSI. Consistently, Li [14]. demonstrated that prolonged operative time significantly increased the incidence of SSI after open reduction and internal fixation of tibial plateau fractures. Therefore, clinicians should closely monitor soft tissue edema in calcaneal fracture patients resulting from high-level falls. Mannitol should be given, and surgery should be delayed to alleviate soft tissue edema. Based on our experience, we recommend 3D CT reconstruction for fractures in this patient population to help surgeons with surgical planning, shorten operation time, and reduce SSI risk.

It has been established that AST is mainly distributed in the myocardium, liver, skeletal muscle, and kidney. In our institution, the normal value of serum AST is 15-40U/L. In this study, AST levels in the SSI group were significantly higher than in the non-SSI group (23.5 versus 21.0 p = 0.038). Consistently, Li et al. [15] demonstrated that AST is a risk factor for SSI after closed tibial plateau fractures. Fractures are widely acknowledged to be accompanied by soft tissue injury. Given that AST is one of the serological markers of muscle injury [16]. AST can reflect the severity of soft tissue injury after fracture to a certain extent. The more severe the peripheral soft tissue injury is, the worse the blood supply, causing failure of the skin of the surgical site to heal in time, thus significantly increasing the risk of SSI [17, 18]. Therefore, we speculate that AST can reflect the degree of soft tissue injury of calcaneal fracture more comprehensively than the macroscopic observation of the fracture site. AST levels should be considered when assessing the degree of soft tissue injury in fracture patients.

Compared with albumin, prealbumin is more sensitive to protein malnutrition and liver dysfunction. It has been reported to play an essential role in physiological processes such as stress response, removal of necrotic material, and tissue repair [19]. In our institution, the normal value range of serum prealbumin is 250–400 mg/L. In this study, prealbumin levels were significantly lower in the SSI group than in the non-SSI group (228.70 versus 262.62 p = 0.001), and prealbumin levels in the SSI group were lower than usual, indicating poor preoperative nutritional status in the SSI group. An increasing body of evidence [20, 21] substantiates low albumin levels as a risk factor for SSI after spinal surgery. Similarly, low prealbumin levels have been reported as a risk factor for SSI in patients with Crohn's disease after intestinal resection [22]. Based on the present study findings and the literature, when the prealbumin value of patients with a calcaneal fracture is lower than usual before surgery, protein supplementation should be increased to improve the nutritional status of patients.

Cystatin C is a cysteine protease inhibitor protein that can better reflect glomerular filtration than BUN and Scr [23]. However, some studies have shown the relationship between cystatin C and infection. Pires et al. [24] demonstrated that cystatin C could treat mycobacterium tuberculosis infection by modulating macrophage immune response. In addition, the study by Pikula et al. [25] showed that cystatin C had good antibacterial activity against Gram-positive bacteria, including multidrug-resistant bacteria, with high safety and could be considered a new antibacterial drug. Besides, in this study, low levels of cystatin C were a risk factor for SSI in calcaneal fractures, and cystatin C levels were significantly lower in the SSI group than in the non-SSI group. To our knowledge, this is the first study to investigate the relationship between cystatin C and SSI. Indeed, more large-scale prospective cohort studies are needed in the future to clarify the association further.

There is ample evidence that the tarsal sinus approach can reduce the risk of SSI in patients with calcaneal fractures compared to the extended lateral approach [26, 27] given that the sinus tarsus approach is a minimally invasive procedure with less dissection of soft tissue, which greatly reduces surgical trauma to patients [28]. However, during statistical analysis in this study, the extended lateral approach was not a risk factor for SSI in patients with calcaneal fractures since the study subjects were patients with unilateral closed comminuted calcaneus fractures, inconsistent with the literature. In addition, in recent years, clinicians have gained a better understanding of SSI and taken corresponding measures to prevent SSI during the perioperative period, which to some extent reduces the risk of SSI in the extended lateral approach.

Few studies have explored the risk factors for postoperative SSI in patients with unilateral closed comminuted calcaneal fractures, and no studies have hitherto visualized these risk factors via a nomogram. Compared with traditional multiple regression models, a nomogram can graphically display all the critical prediction factors. Importantly, our nomogram could help clinicians assess SSI risk in patients with calcaneal fracture during the perioperative period and take necessary measures to prevent SSI (nutritional enhancement, 3D CT reconstruction to shorten the operation time, etc.).

## Limitations

Several limitations were found in this study. First of all, the study's retrospective nature increases susceptibility to selection and recall bias. However, in this study, we minimized the occurrence of these biases by conducting multiple statistical analysis methods. Moreover, the number of patients with postoperative SSI for unilateral comminuted calcaneal fractures was relatively small in this study. Accordingly, multi-center prospective studies with large sample sizes are needed for further study.

## Conclusions

According to our study findings, clinicians should pay more attention to preoperative prealbumin, AST, cystatin C, high-fall injury, and operative time for patients with closed comminuting calcaneal fractures to avoid the occurrence of postoperative SSI. Furthermore, clinicians can use our nomogram model to assess SSI risk in calcaneal fracture patients and take appropriate measures to prevent SSI.

## Data Availability

All data can be obtained from corresponding authors upon reasonable request.

## Abbreviations

BMI: Body mass index
VTE: Venous thrombus embolism
WBC: White blood cell
RBC: Red blood cell
MPV: Mean platelet volume
AST: Aspartate aminotransferase
ALT: Alanine aminotransferase
A/G: Albumin/globulin
CPK: Creatine phosphate kinase
BUN: Blood urea nitrogen
Scr: Serum creatinine
MCHC: Mean corpusular hemoglobin concerntration
ALP: Alkaline phosphatase
GGT: Gamma-glutamyltransferase
FBG: Fasting blood glucose
LDH: Lactate dehydrogenase
MCV: Mean corpuscular volume
PDW: Platelet distribution width
MCH: Mean corpuscular hemoglobin
SSI: Surgical site infection
ROC: Receiver operating characteristic curve
AUC: Area Under Curve
